# Unlocking Bioactive, Peptide-Rich Extracts from Tomato Seeds Using Enzymatic-Assisted Extraction

**DOI:** 10.3390/foods15111934

**Published:** 2026-05-29

**Authors:** Giorgia Benati, Maura Ferri, Tommaso Barbieri, Annalisa Tassoni

**Affiliations:** 1 Department of Biological, Geological, and Environmental Sciences, University of Bologna, Via Irnerio n.42, 40126 Bologna, Italy; giorgia.benati2@unibo.it (G.B.); maura.ferri@unibo.it (M.F.); 2 TomaPaint S.r.L., Strada Canneto-Asola n.46/b, Canneto sull’Oglio, 45013 Mantova, Italy; tommaso.barbieri@tomapaint.it

**Keywords:** bioactive peptides, proteases, sustainable extractions, tomato seeds, anti-tyrosinase activity

## Abstract

Tomato processing generates large amounts of by-products, with seeds representing an underutilized yet protein-rich fraction. This study investigated direct enzyme-assisted protein extraction from non-defatted tomato seeds. Various enzymes, enzyme/substrate ratios, pre-treatments, and incubation temperatures were evaluated and optimized. An enzyme/substrate ratio of 5% (*w*/*w*) was found to be optimal, with proteases alone outperforming cell wall-degrading enzymes and two-step extraction strategies. Bromelain, Protamex, and Trypsin, for the first time applied directly to non-defatted tomato seeds, achieved the highest protein recoveries (average 110.56 mg BSA eq/g DW). Among them, Trypsin also produced the highest reducing sugar content (25.07 mg GLU eq/g DW), indicating effective cell wall disruption. Digestates obtained from defatted and non-defatted tomato seeds showed comparable protein contents, demonstrating that defatting was unnecessary. Avoiding the defatting step improved process sustainability by reducing solvent use and energy consumption without significantly affecting protein extraction efficiency. Incubation at 37 °C was preferred over 60 °C, as similar yields were achieved under milder conditions while also reducing energy consumption by approximately three-fold (54,340 kJ vs 150,480 kJ for a 1000 L water-based scale-up simulation). These digestates showed significantly higher antioxidant and, for the first time in tomato seed extracts, anti-tyrosinase activities compared with controls. Protamex-derived samples exhibited the highest bioactivities (7.40 mg AA eq/g DW; 101.36 μg KA eq/g DW). Compared to conventional alkaline–acid extraction followed by enzymatic digestion, the direct enzymatic approach provided higher protein recovery. Overall, this method represents a sustainable strategy for producing bioactive peptide-rich extracts for food and non-food applications.

## 1. Introduction

Finding alternative protein sources has become essential to meet the dietary needs of the growing world population while mitigating the environmental impact of food production [1,2]. Plant proteins represent a promising alternative to traditional animal-based proteins due to their high nutritional value and health benefits. In particular, agro-industrial by-products represent a valuable resource, as their valorization supports the transition towards more sustainable and healthy diets while reducing food production residues and promoting circular economy principles [2].

Among agro-industrial by-products, tomato processing residues remain an underexploited source of plant proteins. With an estimated global production of 188 million tons in 2024, the tomato is among the most cultivated crops. Europe alone accounted for approximately 22.5 million tons in 2024, of which 26.6% (about 6 million tons) was cultivated in Italy [3]. The tomato processing industry includes the production of sauces, pastes, canned tomatoes, purees and other related products, generating a substantial amount of waste (2–5% *w*/*w* of raw tomatoes), mostly consisting of tomato pomace (peels, seeds and residual pulp) [4]. Although rich in nutrients and a source of bioactive compounds, such by-products are still predominantly used as animal feed [4] or disposed of in landfills, resulting in ecological issues [5].

Many studies have investigated the potential applications of tomato pomace and tomato peels, whereas tomato seeds (TSs), despite representing approximately 60% of tomato pomace and being rich in added-value compounds, still remain insufficiently valorized [6]. Their main constituents are oils (17.8–24.5% dry weight) and proteins (20.2–40.9% dry weight), but they also contain fibers (16% dry weight) [5]. Specifically, TSs represent a valuable source of high-quality proteins, due to their content of globulins, albumins, prolamines and glutelines [7]. Glutamic acid is the most present amino acid, followed by aspartic acid. In addition, TSs are a good source of essential amino acids as they contain arginine, threonine, lysine, and leucine, up to 39.5% of the total [5,6]. The higher content of lysine compared to other plant proteins is also a distinctive advantage of TS proteins [8]. Although most efforts have targeted the extraction of oils [9], the recovery of proteins from this feedstock could represent a promising opportunity for its valorization, especially when achieved through environmentally sustainable extraction methods.

Conventionally, protein extraction from plant sources is carried out via alkaline solubilization followed by acidic precipitation at the isoelectric point, including for tomato-derived residues such as TSs [10,11]. Although this approach has been widely adopted, it presents several drawbacks. Extreme alkaline conditions can cause protein denaturation, induce amino acid racemization, and also cause the formation of lysinoalanine cross-links, which can impair protein functional properties and digestibility and reduce nutritional value [12]. Beyond these limitations, this process often leads to salt formation, which is generally undesirable and requires extensive use of bases and acids, potentially raising environmental concerns [12,13].

Enzyme-assisted extraction represents a promising, eco-friendly alternative that is increasingly applied to recover proteins from plant material and agricultural residues [1,2]. A major advantage of this approach is that enzymes operate under milder conditions than conventional methods, enabling extraction at near-neutral pH and thereby preventing protein damage and the consequent loss of functional properties and nutritional value [12]. Additional benefits include lower energy requirements, minimized waste generation [1], reduced consumption of chemicals and toxic solvents due to water-based processing [14], and reduced salt formation [12].

More importantly, enzyme-assisted extraction generates low-molecular-weight peptides (2 to 20 amino acids). These peptides, produced through the selective cleavage of peptide bonds, often exhibit a wide range of bioactivities that are generally lower or absent in their precursor proteins [15,16]. Consequently, the resulting extract may exhibit enhanced functional properties [17], along with improved peptide bioavailability for human consumption [2].

In this work, different proteases and cell wall hydrolytic enzymes were applied to tomato seeds separated from tomato pomace, either individually or in combination, to optimize protein extraction. In addition, various processing parameters and pre-treatments were evaluated to identify the most efficient and sustainable recovery process. The protein profiles of the resulting digestates were characterized, and their antioxidant and anti-tyrosinase activities were assessed. While enzymatic treatment is typically performed on protein isolates obtained after alkaline extraction and isoelectric precipitation [11], to the best of the author’s knowledge, this study is the first to explore both proteases and cell wall hydrolytic enzymes directly on raw tomato seeds, avoiding defatting and optimizing the extraction protocol with mild incubation temperatures to reduce environmental and energy costs. To evaluate potential differences in the yield and characteristics of the recovered proteins, conventional alkaline–acid and neutral–acid extraction methods followed by enzymatic digestion were also employed for comparison. By enabling the sustainable recovery of proteins and bioactive peptides from tomato seeds, this study seeks to address the gaps for their valorization and contribute to a more sustainable agricultural system within the framework of the circular economy.

## 2. Materials and Methods

### 2.1. Raw Material

Tomato seeds (TSs), obtained from tomato pomace and separated from peels using a flotation system, were provided by TomaPaint S.r.L. (Mantova, Italy). The flotation system consisted of a loading area and a separation area. Tomato pomace (2500 kg/h) was introduced into a separation tank (10 m^3^ capacity) operating by flotation to separate skins from seeds. Due to differences in specific weight, skins floated on the water surface (30 °C), while seeds settled at the bottom. Water (1000 L/h) was continuously supplied to facilitate the sedimentation of seeds trapped among the skins. Approximately 1540 kg/h of seeds were collected from the bottom of the flotator using a centrifugal pump and transferred to a rotary filter to separate the seeds from the liquid fraction. The seeds were oven-dried overnight at 80 °C, due to the necessity of ensuring rapid moisture removal to avoid spoilage, until the weight was constant [18]. The fresh weight (FW) of the seeds was recorded before drying, while the dry weight (DW) was obtained by re-weighting them after drying. The DW of the TSs accounted for 24.6% ± 0.9% of the FW. Seeds were then ground with an electrical mill (IKA A11 basic, Staufen im Breisgau, Germany), and the resulting TSs powder was stored at 4 °C. Protein content of TSs was determined using the Kjeldahl method [19].

### 2.2. Enzyme-Assisted Protein Extraction

Enzyme-assisted protein extraction was performed by using seven commercial proteases namely Alcalase^®^ from *Bacillus licheniformis* (Sigma-Aldrich, Milan, Italy), bromelain from pineapple stem (Sigma-Aldrich, Milan, Italy), Neutrase^®^ (Novozymes A/S, Bagsværd, Denmark), pancreatin from porcine pancreas (Sigma-Aldrich, Milan, Italy), papain from *Carica papaya* (Sigma-Aldrich, Milan, Italy), Protamex^®^ from *Bacillus* sp. (Novozymes A/S, Denmark), trypsin from porcine pancreas (Sigma-Aldrich, Milan, Italy). The selected enzymes represent different types of endopeptidases, with the exception of pancreatin, which is a mixture of digestive enzymes exhibiting both endopeptidase and exopeptidase activities.

Additionally five plant cell wall hydrolytic enzymes were selected based on their ability to digest different components of the plant cell wall: cellulase from *Trichoderma reesei* (Celluclast^®^, Sigma-Aldrich, Milan, Italy), pectinase from *Aspergillus aculeatus* (Pectinex^®^ Ultra SP-L, Sigma-Aldrich, Milan, Italy), pectinase from *Aspergillus niger* (Pectinex^®^ XXL, Sigma-Aldrich, Milan, Italy), xylanase from *Thermomyces lanuginosus* (Pentopan^®^, Sigma-Aldrich, Milan, Italy) and Viscozyme^®^ L, a multienzyme cocktail capable of hydrolyzing several plant cell wall polysaccharides (Sigma-Aldrich, Milan, Italy). Milli-Q water was added to TSs (2 gDW of ground TSs) with a solid/liquid ratio (S/L) of 1:5. For individual enzyme digestions, each enzyme was added separately at ratios corresponding of 1%, 2% or 5% enzyme to substrate (E/S, *w*/*w*) ratio and the pH was adjusted with 1 N NaOH to 7.0 ± 0.2 or to 6.0 ± 0.2 for protease or cell wall hydrolytic enzyme treatments, respectively according to manufacturer’s instructions. The samples were incubated for 2 h either at 60 °C (for the first trials) or 37 °C (for the following optimization trials) and kept shaking at 100 rpm. Subsequently, for combined two-step enzyme digestions, Viscozyme was selected as the most effective cell wall hydrolytic enzyme, and digestion was carried out consecutively: Viscozyme treatment at pH 6.0 followed by protease treatment at pH 7.0, for a total incubation time of 4 h. After each incubation, samples were placed in boiling water for 10 min to inhibit enzyme activity. Then, they were centrifuged (5000 rpm, 20 min, room temperature (RT)) to separate the solid residue (pellet), the lipid floating fraction (if present), and the liquid fraction (digestate), which was used for all subsequent analysis. Samples included non-digested controls (ND), obtained by adding water followed by immediate centrifugation, and thermally digested controls (TD), which were processed under the same conditions as enzyme-treated samples but without enzyme addition. For the TD, the pH was adjusted to 7.0 ± 0.2 for comparison with protease treatments or to 6.0 ± 0.2 for comparison with cell wall hydrolytic enzyme treatments. After centrifugation, all digestates were stored at −20 °C until further analysis.

A defatting pre-treatment was also tested: dried TSs were incubated with 100% ethyl acetate (S/L 1:10 *w*/*v*) and stirred for 30 min at RT. After centrifugation (5000 rpm, 10 min, RT), seeds were recovered, and residual ethyl acetate was removed by allowing it to evaporate under a fume hood. The defatted tomato seeds (dTSs) were subsequently ground and used for enzyme-assisted protein extraction.

To estimate the energy consumption of a potential industrial pilot-plant scale-up, the heat transfer equation [20] was applied to calculate the energy required to heat a water-based solution in a 1000 L pilot bioreactor from 24 °C to either 60 °C or 37 °C.

$$Q=m\times c_{p}\times \Delta T$$

where

*Q* is the energy required (kJ);
*m* is the mass of the extraction volume (kg);
*c_p_* is the specific heat capacity of the substance (i.e., for water 4.187 kJ kg^−1^ °C^−1^);
Δ*T* is the temperature difference, corresponding to heating from 24 °C to 60 °C or from 24 °C to 37 °C.

### 2.3. Digestate Characterization

Digestates were analyzed to assess total amounts of proteins [21], reducing sugars [22], and phenols [23]. All methods used external standards, respectively bovine serum albumin (BSA), D-glucose (GLU) and gallic acid (GA), by means of dose–response calibration curves: 0–200 µg for BSA, 50–500 µg for GLU, and 0–15 µg for GA. The results were expressed as mg of equivalent standard per g of TS dry weight (mg eq/gDW). Since these colorimetric assays were applied to complex biological extracts, their specificity may have been potentially influenced by matrix interferences and the presence of unpredictable non-target reactive compounds [24,25].

SDS-PAGE was performed on digestates and their ND and TD controls using Mini-PROTEAN^®^ TGX Precast Protein Gels (BioRad, Irvine, CA, USA) with an 8–16% polyacrylamide gradient [26]. Each sample (25 to 10 μL of sample) was mixed with 5 μL of sample buffer (SB, 62.5 mM Tris-HCl at pH 6.8, 2% (*w*/*v*) SDS, 25% (*v*/*v*) glycerol, 0.01% (*v*/*v*) bromophenol blue, 100 mM 1,4-dithiothreitol added fresh) and denatured in boiling water for 5 min. For molecular weight determination, 4 μL of protein markers with ranges of 8.0–220 kDa and 1.0–26.6 kDa (Sigma-Aldrich, Milano, Italy) were equally processed and loaded. Gels were stained with Bio-Safe Coomassie G-250 Stain (BioRad, Irvine, CA, USA) following the manufacturer’s instructions.

### 2.4. Biological Activities Assays

The antioxidant and anti-tyrosinase properties of selected digestates and controls were evaluated through the ABTS (2,2-azino-bis-3-ethylbenzothiazoline-6-sulfonic acid) and the anti-tyrosinase assays, following the procedure reported in Ferri et al. [27]. Results were expressed respectively as mg of ascorbic acid (AA) equivalents and as µg of kojic acid (KA) equivalents per gram of TS dry weight (g DW) by means of dose–response calibration curves (0–2 µg for AA and 1–10 µg for KA).

### 2.5. Alkaline–Acid and Neutral–Acid Protein Extraction Followed by Enzymatic Digestion

TSs (2 gDW) were incubated at a S/L ratio of 1:5 either with 0.1 M NaOH (pH 11) or 0.05 M Na_3_PO_4_ and 0.1 M NaCl (pH 7.2). Incubation was carried out at 24 °C for 3 h under constant agitation (120 rpm). Following incubation, samples were centrifuged at 5000 rpm for 20 min at RT, and the solid residue was separated from the supernatant containing the solubilized proteins. Protein precipitation in acidic conditions (pH 3) was achieved using 6 N HCl in the case of alkaline solubilization, or 85% (*v*/*v*) H_3_PO_4_ in the case of neutral solubilization. The suspensions were centrifuged again at 5000 rpm, 20 min, 4 °C, and the protein pellet and the supernatant were separated and collected. Both fractions were analyzed for protein content [21] after resuspending the protein pellet in 0.05 M Na_3_PO_4_ added with 0.1 M NaCl (pH 7.2 ± 0.1) at an average concentration of 0.1 gFW/mL. The DW of the protein pellet accounted for 24.1% ± 2.1% of the FW after drying overnight at 80 °C.

Subsequently, the alkaline/acid process was scaled up to a final volume of 400 mL to obtain enough protein isolate to perform enzymatic digestions. The pellet was subjected to several washes with Milli-Q water (1:6 S/L ratio, until weight was constant) to eliminate the presence of salts and weighed to establish the FW. Each enzymatic digestion was carried out at 60 °C for 2 h with Bromelain, Protamex or Trypsin (5% (*w*/*w*) E/S) on roughly 2 g FW of TS protein isolate as described in Section 2.2. ND and TD controls were also performed. Protein content of digestates was determined [21] and results expressed as mg BSA eq/g DW.

### 2.6. Statistical Analysis

All extractions were performed in four biological replicates, each one analyzed in technical duplicates. Statistical analysis was performed using R (version 4.3.2) [28]. Variance among samples was assessed by ANOVA test followed by post hoc Tukey HSD (package *agricolae*) [29]. Differences were considered significant with a *p*-value < 0.05.

## 3. Results and Discussion

### 3.1. Protein Quantification in Raw Material

The protein content of raw tomato seeds (TSs), determined by Kjeldahl analysis, was 26.1% ± 0.7%. This value falls within the range determined in previous studies and is comparable to those reported by Baker et al. [8] (27.4%) and by Maldonado-Torres et al. [30] (approximately 26.9%). The protein content obtained in the present study is slightly higher than that reported by Mechmeche et al. [7] (23.6%), but lower than the value reported by Fuentes et al. [31] (32%). These variations may be attributed to differences in tomato cultivar, growing conditions, seed maturity, and post-harvest processing.

### 3.2. Individual Enzyme Trials

**Figure 1:**
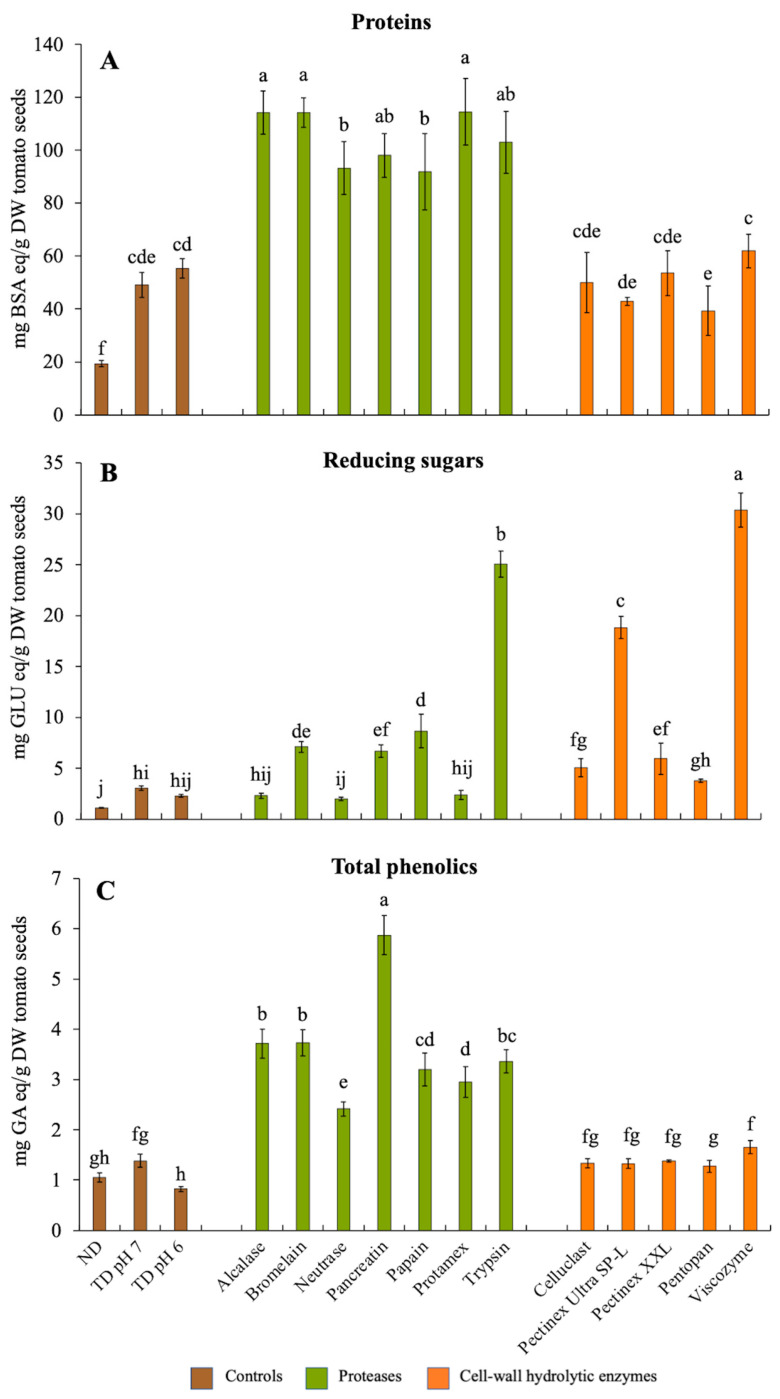
Content of (**A**) proteins (mg BSA eq/g DW), (**B**) reducing sugars (mg GLU eq/gDW), and (**C**) total phenolic compounds (mg GA eq/gDW) in tomato seed digestates after treatment with different proteases and cell wall hydrolytic enzymes (5% *w*/*w* E/S ratio) at 60 °C for 2 h. Letters indicate statistically significant difference among samples determined by ANOVA test followed by post hoc Tukey HSD test (*p* < 0.05). BSA, bovine serum albumin; DW, dry weight; GLU, glucose; GA, gallic acid; ND, non-digested control; TD, thermally digested control. Data are the mean (n = 4) ± SD.

The initial optimization phase focused on the determination of the best enzyme-to-substrate ratio (E/S) and the most efficient enzymes for extracting proteins and peptides from TSs. TSs were digested with proteases and cell wall hydrolases added individually at E/S ratios corresponding to 1%, 2% or 5% (*w*/*w*) (Supplementary Table S1, Figure 1).

Although some authors reported that increasing enzyme dosage did not significantly impact protein extraction [13,32], in the present study, higher E/S ratios generally led to an increase in the analyzed compounds in the digestates, although the response varied depending on the type of enzyme and compound. Specifically, protein release increased as the E/S ratios of proteases were raised; reducing sugar levels were generally higher with increasing E/S ratios of most proteases and all cell wall hydrolytic enzymes. In contrast, an increase in phenolic compounds was observed only at the 5% (*w*/*w*) E/S ratio of Bromelain, Pancreatin, and Papain.

No specific differences in protein content were observed between 1% and 2% (*w*/*w*) E/S for protease treatments, except for Trypsin (Supplementary Table S1). Alcalase, Bromelain, Neutrase, Pancreatin and Protamex exhibited significantly higher efficiency in releasing proteins from TSs at 5% (*w*/*w*) E/S compared with 1% and 2% (*w*/*w*) E/S, with average increases of 32.5% and 21.4%, respectively. In contrast, Papain- and Trypsin-treated samples showed comparable protein content at 2% and 5% (*w*/*w*) E/S, although both were still significantly higher than those obtained at 1% E/S (average increase of 36.7%) (Figure 1A, Supplementary Table S1).

Among the cell wall hydrolytic enzymes, in the present digestion conditions, only Viscozyme-treated samples displayed a significant increase in protein content at 5% (*w*/*w*) E/S compared to other dosages, with increases of 32.6% respect to 1% E/S and of 29.2% respect to 2% E/S (Figure 1A, Supplementary Table S1).

All proteases were able to enhance protein release compared to both non-digested (ND) and thermally digested (TD, pH 7) controls by an average of 5.4-fold and 2.1-fold, respectively, at 5% (*w*/*w*) E/S. Among them, Alcalase, Bromelain, Protamex, and, to a lesser extent, Trypsin released the highest protein contents in the digestates, with values up to 114.5 mg BSA eq/gDW in Protamex 5% (*w*/*w*) E/S treated samples (Figure 1A). In contrast, plant cell wall hydrolytic enzymes did not exhibit a notable improvement in protein release compared to the TD (pH 6) control under the tested conditions. Viscozyme at 5% (*w*/*w*) E/S released the highest amount of proteins (61.9 mg BSA eq/g DW) (Figure 1A).

Increasing enzyme dosage has been widely reported to enhance protein yield during enzymatic extraction across different plant matrices [1], consistent with the results of this study (Figure 1A, Supplementary Table S1). Higher Alcalase and Viscozyme dosages have been shown to improve protein recovery from brewery spent grain and okara, respectively, although excessive enzyme addition often leads to a plateau or decline in yield [33,34]. In contrast, Hanmoungjai et al. [35] found that increased enzyme levels improved protein extraction from rice bran with Alcalase but not Papain, mirroring the trends observed in the present study.

Although some studies have reported that cell wall hydrolytic enzymes can increase protein yield compared to controls in various plant biomasses (e.g., defatted soy flour) [36], the results obtained for tomato residues in this study are consistent with previous literature indicating that proteases are more effective for protein recovery. To date, very few studies have applied enzyme-assisted extraction to raw tomato seeds, and none have investigated proteases on non-defatted seeds. In this context, the present work provides several advances. Zhang et al. [37], working on defatted tomato seed meal (i.e., the solid protein-rich residue obtained after hexane-based oil extraction from tomato seeds), which differs from the matrix used in this study due to the removal of lipids with solvent extraction, reported enhanced protein content especially in lower particle size fractions following Papain treatment, with a primary focus on umami-related amino acids (glutamic and aspartic acids). In contrast, the present study evaluated a broader range of enzymes applied to raw and non-defatted TSs, confirming the superior performance of proteases over cell wall hydrolytic enzymes and identifying proteases (Alcalase, Bromelain, Protamex, and Trypsin) that outperform Papain in protein recovery (Figure 1A). Baker et al. [8] investigated non-defatted freeze-dried cherry tomato seeds but did not evaluate proteases for protein recovery. In that study, cell wall hydrolytic enzymes applied directly to tomato seeds were largely ineffective; only Filta 02L produced a modest increase in protein yield, while the other enzyme formulations tested, although different from those used here, did not improve protein recovery compared to the control.

While cell wall hydrolytic enzymes showed limited success in improving protein yield, they were, as expected, more successful than proteases in degrading the cell wall, as reflected by the higher levels of reducing sugars in the digestates (Figure 1B, Supplementary Table S1). Among them, Viscozyme at 5% (*w*/*w*) E/S exhibited the strongest effect compared with both ND and TD controls, reaching maximum values of 30.38 mg GLU eq/g DW, followed by Pectinex Ultra SP-L (18.83 mg GLU eq/g DW). Some proteases also promoted reducing sugar release, with the best results observed in samples treated with 5% (*w*/*w*) E/S Trypsin, which reached up to 25.07 mg GLU eq/g DW (Figure 1B). Overall, cell wall hydrolytic enzymes showed a higher capacity to enhance reducing sugar release at 5% (*w*/*w*) E/S (Figure 1B) compared with lower enzyme ratios, exhibiting an average 2.0-fold increase with respect to 1% (*w*/*w*) E/S and a 1.7-fold increase with respect to 2% (*w*/*w*) E/S (Supplementary Table S1). In contrast, only four proteases (i.e., Bromelain, Pancreatin, Papain, and Trypsin) treatments showed increased release of reducing sugars with increasing E/S ratios.

To the best of our knowledge, no previous studies have specifically investigated reducing sugar release from tomato seeds following enzymatic treatment. Although Baker et al. [8] applied cell wall hydrolytic enzymes to cherry tomato seeds for protein recovery, reducing sugar content was not assessed. On the other hand, comparable results in terms of reducing sugar release have been reported for different biomasses, such as rice bran, particularly following enzymatic treatment with Viscozyme and Pectinex Ultra SP-L, which released approximately 11.5- and 7.5-fold more reducing sugars than the respective controls [35]. In that study, neither of the tested proteases (Alcalase and Papain) increased reducing sugar release. This finding is consistent with the results observed here for Alcalase but in contrast with those for Papain, which instead proved to be more effective (Figure 1B).

Proteases, unlike the cell wall hydrolytic enzymes, were also effective in releasing significantly higher amounts of phenols compared to the ND and TD (3.4-fold and 2.6-fold increases, respectively), particularly when increasing enzyme dosages of Bromelain, Pancreatin, and Papain were applied (Figure 1C, Supplementary Table S1). Among 5% (*w*/*w*) E/S ratio protease digestates, Pancreatin-treated samples showed the highest content of total phenolics (5.87 mg GA eq/g DW), averaging 1.9-fold higher than other protease digestates (Figure 1C). Pancreatin is a mix of enzymes, namely proteases, lipases and glucosidases; therefore, the enhanced release of phenolics may result from their combined action. Cell wall hydrolytic enzymes did not extract significantly higher amounts of total phenolics compared to ND and TD controls, and to protease treatments. Although tomato seeds generally contain lower levels of phenolics compared to tomato peels, they could represent a good source of gallic and ferulic acids [38]. Previous papers reported a total phenolic content of 0.10–0.12 mg GA eq/g [31]. However, to the best of the authors’ knowledge, no studies have attempted the enzymatic extraction of phenolic compounds from TS, and extractions are usually made with organic solvents like ethanol and methanol or with water [38].

Due to their higher efficiency in protein recovery, the proteases Bromelain, Protamex and Trypsin at 5% (*w*/*w*) E/S ratio were selected for the following experiments. Although Alcalase also released a significant amount of proteins (Figure 1A), it was excluded based on previous evidence indicating its low cleavage-site specificity, which can lead to over-digestion of the released peptides and, consequently, loss of functionality [11,27]. The choice of these three proteases also allows to compare enzymes of different origins: Bromelain is obtained from pineapple, representing a plant protease; Protamex, a mixture of endopeptidases produced by *Bacillus* sp., is microbial; while Trypsin, obtained from porcine pancreas, is of animal origin. Among the plant cell wall hydrolases tested, Viscozyme at 5% (*w*/*w*) E/S was selected due to its slightly higher efficiency in protein extraction compared with the other cell wall-degrading enzymes (Figure 1A), as well as its greater capacity to release reducing sugars (Figure 1B). The latter indicates more effective cell wall disruption.

### 3.3. Two-Step Combined Enzyme Trials

A two-step digestion approach was investigated to determine whether a first step with Viscozyme (an enzymatic cocktail comprising cellulase, hemicellulase, xylanase, arabinase, and β-glucanase) could enhance protein recovery by increasing protease access to the matrix during the subsequent protease treatment.

The addition of proteases after Viscozyme (both at 5% *w*/*w* E/S ratios) resulted in an increased protein yield compared to both Viscozyme-only digestion (Figure 1A) and to TD control (4 h, Table 1) by an average of 1.7-fold and 1.9-fold, respectively. However, the preliminary treatment with Viscozyme did not further enhance protein recovery beyond the use of proteases alone, with an average of 104.06 mg BSA eq/g DW in two-step combined digestion and an average of 110.56 mg BSA eq/g DW in single digestions with 5% (*w*/*w*) E/S of selected proteases (Table 1, Figure 1A). Reducing sugars and total phenols contents in two-step digestion samples were on average 4.6-fold and 1.9-fold higher than those detected in single proteases and Viscozyme treatments (Table 1, Figure 1B,C) as a result of the combined action of the enzymes.

To date, no studies on tomato processing residues have reported the use of a two-step enzyme-assisted extraction. In other biomasses, results remain inconsistent: while the combination of cell wall-degrading enzymes and proteases has been shown to enhance protein recovery in some cases [32], other studies reported no improvement over protease treatment alone [12,35].

Considering these results and to improve the environmental and economic sustainability of the extraction process (reducing time, energy, and additional enzyme costs), it was decided to use only proteases for the extraction of proteins from TS samples.

### 3.4. Further Optimization Tests

Further optimization tests were conducted only with a few selected proteases to further improve protein extraction beyond initial results.

#### 3.4.1. Defatting Trials

Evidence from various biomasses, among which tomato seed meal, indicated that defatting improves alkaline/acid protein extraction by removing interfering oils [12,30,39,40]. Based on this evidence, the enzyme-assisted extraction protocol was applied to defatted tomato seeds (dTSs) to assess whether this pre-treatment could further improve protein extraction beyond initial results. Supporting this approach, Sari et al. [13] reported lower protein extraction yields from non-defatted microalgae compared with defatted samples following protease treatment, suggesting that defatting can enhance protein recovery even when enzymatic methods are employed.

However, in this study, defatting did not confer a significant advantage (Supplementary Table S2). Protein content in protease-treated samples obtained from dTSs was not significantly higher than that of non-defatted tomato seeds (TSs). Specifically, the average protein yield of Protamex and Trypsin-treated samples was 108.73 mg BSA eq/g DW for TSs (Figure 1A) and 111.27 mg BSA eq/g DW for dTSs (Supplementary Table S2), corresponding to a statistically non-significant 2.3% increase. In previous studies involving enzymatic digestion of TSs, Zhang et al. [37] applied Papain to hexane-defatted tomato seed meal but did not evaluate non-defatted TSs.

Given the minor and non-significant improvement in protein recovery, the use of non-defatted TSs was deemed the most sustainable option, as it reduces processing time, solvent use, and operational costs, particularly considering the special handling requirements of ethyl acetate. Indeed, besides eliminating the cost of the solvent used for defatting, in pilot scale studies, organic solvents present flammability issues as well as increased CAPEX (CAPital Expenditure) and OPEX (Operating Expenditure) [8].

#### 3.4.2. Incubation Temperature

To further reduce the environmental impact of the enzymatic extraction process, the extraction incubation temperature was lowered from 60 °C to 37 °C, while all other parameters remained unchanged. The selected temperature falls within the working range of the tested Bromelain and Protamex proteases, and is actually optimal for Trypsin instead of the 60 °C.

Several studies highlighted how excessively low and excessively high temperatures resulted in reduced protein recovery. For instance, enzymatic extractions from quinoa conducted at different temperatures reported a gradual increase in protein extraction yield up to 50 °C, followed by a decline at 60 °C [41]. Previous reports further indicate that higher temperatures can exert both positive and negative effects: while they may enhance protein solubility by softening tissues and improving extraction efficiency, they can also promote thermal degradation, potentially compromising protein integrity and bioactive properties [1].

**Figure 2:**
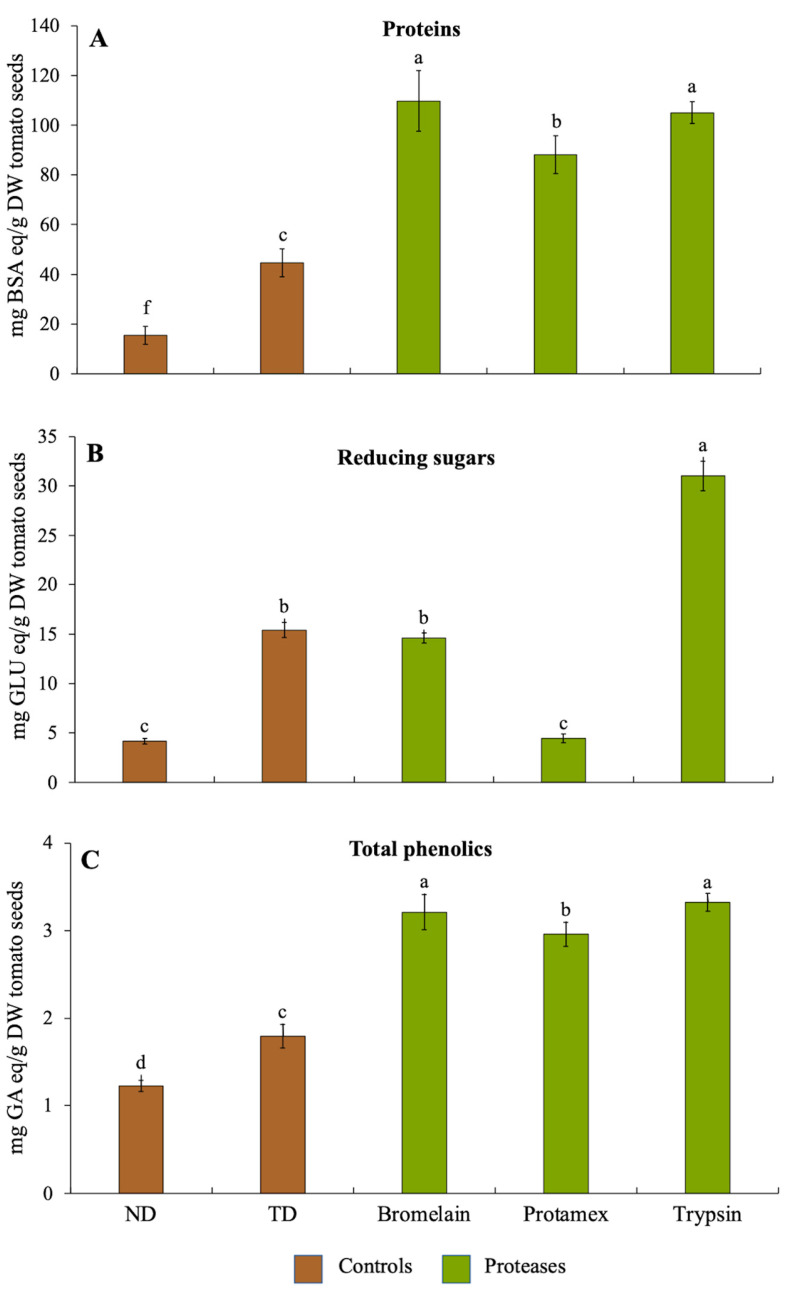
Content of (**A**) proteins (mg BSA eq/g DW), (**B**) reducing sugars (mg GLU eq/g DW), and (**C**) phenolic compounds (mg GA eq/g DW) in tomato seeds digestates following enzymatic digestion at 37 °C for 2 h with selected proteases. Letters indicate statistically significant difference among samples determined by ANOVA test followed by post hoc Tukey HSD test (*p* < 0.05). BSA, bovine serum albumin; DW, dry weight; GA, gallic acid; GLU, glucose; ND, not-digested control; TD, thermally digested control. Data are the mean (n = 4) ± SD.

Overall, 37 °C protease-treated digestates exhibited a higher protein content compared to the ND and TD, with average increases of 6.5-fold and 2.3-fold, respectively (Figure 2). TD at the two incubation temperatures were statistically similar (Figure 1A and Figure 2). Protein concentrations in the digestates were statistically comparable between incubations at 60 °C (Figure 1A) and 37 °C (Figure 2), with the only exception of Protamex-treated samples, which showed about 23% lower protein content at 37 °C compared with 60 °C. Overall, the differences between extractions at 37 °C and 60 °C were limited, indicating that incubation temperature generally did not significantly affect protein recovery from TSs.

Besides preserving protein integrity, operating at a lower incubation temperature also reduces energy consumption. A simulation based on a 1000 L industrial pilot-scale extraction estimated the energy required to heat the system from 24 °C to either 37 °C or to 60 °C. The results indicated that heating to 37 °C would require approximately three times less energy (54,340 kJ) than heating to 60 °C (150,480 kJ). Although simplified, as the calculation considered only the specific heat capacity of the water fraction, these estimates may provide an initial indication of the different energy demands associated with the two incubation temperatures. Thus, the lower temperature appears more advantageous for a potential scale-up, as it reduces the risk of protein degradation while also lowering the energy consumption and environmental impact of the process, while maintaining comparable protein yields. This improvement also represents a further innovation compared with previous reports on TSs and defatted TSs meal, where enzymatic hydrolysis was performed at 50–54 °C [8,37].

Reducing sugar release was significantly higher at 37 °C (Figure 2B) than at 60 °C (Figure 1B), with protease-treated samples showing an average 1.7-fold increase. The relative distribution of reducing sugars among treatments mirrored the pattern observed at 60 °C. Trypsin-treated samples exhibited the highest levels, reaching up to 31.01 mg GLU eq/g DW. Bromelain-treated samples showed intermediate values, comparable to the TD control at 37 °C. In contrast, Protamex-treated samples displayed the lowest reducing sugar content, which was statistically similar to the ND control (Figure 2B). On the other hand, total phenolic content was generally comparable between 37 °C and 60 °C incubations, with the exception of Bromelain-treated samples, which were 1.2-fold higher at 60 °C (Figure 1C and Figure 2C). Similar to reducing sugars, the distribution of total phenols across treatments at 37 °C followed the pattern observed at 60 °C, with Bromelain- and Trypsin-treated samples showing the highest levels (3.21 and 3.32 mg GA eq/g DW, respectively) (Figure 2C). Previous studies [42] have employed cell wall-degrading enzymes as a pre-treatment step to extract phenol-rich oleoresins from tomato waste (peels and seeds). However, those incubations were conducted at 45 °C or 50 °C, which are still higher than the optimized temperature used in the present study.

### 3.5. Molecular and Functional Characterization of Extracts

Mono-dimensional SDS-PAGE was performed on enzyme digestates at 37 °C and related ND and TD controls, and revealed the presence of low-molecular-weight peptides (<12 kDa) (Supplementary Figure S1). Besides enzyme digestates, ND and TD samples also exhibited low-molecular-weight peptides. However, these peptides may have been generated in tomato seeds during industrial tomato processing and subsequently released into the liquid supernatant during the extraction incubation step. In the TD control, thermal treatment may have further promoted partial protein breakdown and facilitated peptide solubilization. Unlike thermal digestion, which is non-specific and produces a heterogeneous peptide mixture with unpredictable composition, protease treatments may provide greater control over the resulting peptides, with specific cleavage sites of peptide bonds [14].

**Figure 3:**
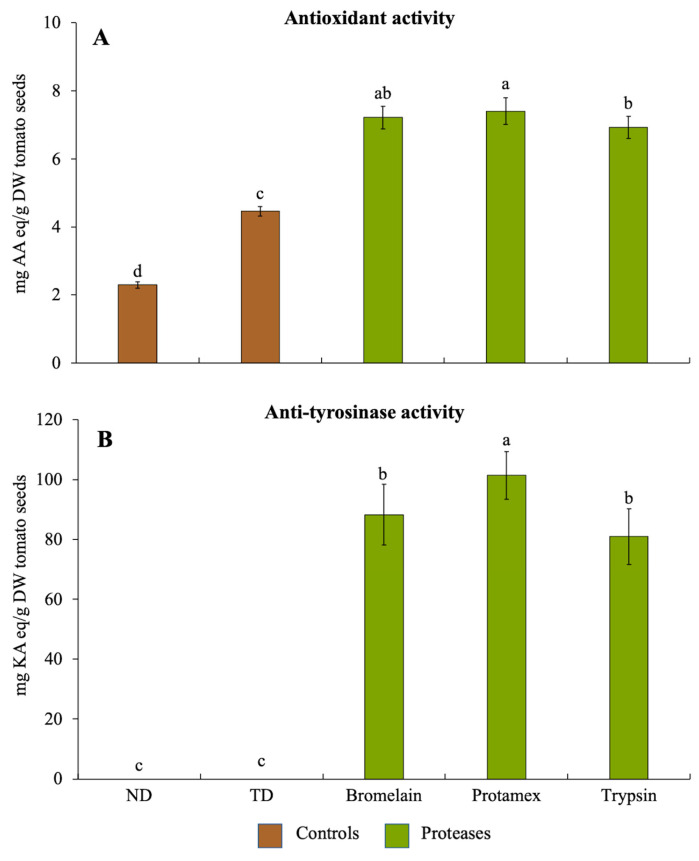
Antioxidant activity (mg AA eq/gDW) (**A**) and anti-tyrosinase activity (μg KA eq/gDW) (**B**) in supernatants following enzyme-assisted protein extraction at 37 °C for 2 h. Letters indicate statistically significant difference among samples determined by ANOVA test followed by post hoc Tukey HSD test (*p* < 0.05). AA, ascorbic acid; DW, dry weight; KA, kojic acid; ND, non-digested control; TD, thermally digested control. Data are the mean (n = 4) ± SD.

Low-molecular-weight peptides generated through protease treatment are known to exhibit bioactivities [17]. Therefore, digestates obtained by enzyme-assisted extraction at 37 °C, along with their corresponding controls (Figure 2 and Figure S1), were evaluated for antioxidant activity (ABTS assay) and anti-tyrosinase activity. In both assays, protease-digested samples exhibited significantly higher bioactivity compared to both ND and TD controls (Figure 3). These results support the hypothesis that, although ND and TD samples contained peptides, those generated through targeted protease treatments were more specific and displayed enhanced bioactivity.

Regarding antioxidant activity, protease-treated samples showed, on average, a 3.1-fold increase compared to the ND control and a 1.6-fold increase relative to the TD control (Figure 3A). Protamex generated the highest antioxidant activity, reaching 7.40 mg AA eq/gDW, followed by Bromelain with statistically comparable values. Although other compounds present in the enzyme-based digestates may have marginally contributed to the observed activity [30], the significantly higher antioxidant capacity of the protease-digested samples compared to the TD control suggested that a large part of this bioactivity could be attributable to peptides generated by enzymatic action.

A previous study reported antioxidant properties of TS proteins/peptides extracted through neutral extraction followed by acidic precipitation [7]. In that work, extracts obtained from defatted tomato seed meal exhibited antioxidant activity in both DPPH and ABTS assays. In contrast, the present study generated bioactive peptides using a more sustainable extraction, as the seeds were not defatted and protease digestion was applied directly to TS, thereby avoiding the protein isolation step involving alkaline solubilization and acidic precipitation.

Regarding anti-tyrosinase activity, neither the ND nor the TD control showed detectable activity. In contrast, protease-treated samples exhibited measurable anti-tyrosinase activity with an average value of 90.17 μg KA eq/g DW, confirming the specificity of protease cleavage. Among the tested proteases, Protamex-treated samples again displayed the highest activity, reaching 101.36 μg KA eq/g DW, which was significantly higher with respect to other digestates (Figure 3B).

The increased anti-tyrosinase activity observed in protease-treated samples could be attributed to the specific action of Bromelain, Protamex, and Trypsin, which generate distinct peptide profiles due to their different cleavage specificities. Bromelain has broad specificity and preferentially cleaves peptide bonds near hydrophobic or aromatic amino acids such as Leu, Ala, Phe, and Tyr, promoting the release of peptides that may interact with the tyrosinase active site or chelate the copper ions involved in enzyme activity [43]. Tyrosinase-inhibitory peptides were obtained from rice starch by-products hydrolyzed with Protamex and containing Ser, Thr, and Tyr residues capable of interacting with the enzyme active site through hydrogen bonding [16]. Trypsin cleaves near positively charged Lys and Arg residues, which may also contribute to tyrosinase inhibition [44].

To the best of the authors’ knowledge, this is the first study reporting the anti-tyrosinase activity of peptides generated directly from non-defatted TS via enzyme-assisted extraction. Existing research has instead focused on other tomato matrices; for example, Kamkaen et al. [45] and Han and Gong [46] evaluated the anti-tyrosinase activity of whole tomato fruit and tomato pulp, respectively, showing higher activity in the pulp. Although the anti-tyrosinase activity of TS has not been previously investigated, other biological activities have been reported. Kartal et al. [47] conducted an in silico analysis of seven TS proteins and predicted that enzymatic hydrolysis could release peptides with ACE-inhibitory, DPP-IV-inhibitory, and antioxidant activities. In that study, Bromelain, along with combinations of Trypsin and Pepsin or Pepsin and Chymotrypsin, were predicted to generate a high frequency of bioactive peptides. Among the three proteases tested, Protamex consistently demonstrated the highest efficiency bioactive peptides (Figure 3). Beyond its superior performance, the use of Protamex presents clear sustainability advantages. It consists of a blend of endopeptidases produced by *Bacillus* sp., and its microbial origin enables cost-effective, scalable, and resource-efficient manufacturing. Consequently, Protamex represents a more sustainable option compared with proteases derived from animal or plant sources [2].

Although the in vitro antioxidant and anti-tyrosinase activities do not necessarily reflect in vivo conditions, the results nevertheless demonstrate the significant bioactive potential of the protease-treated samples.

### 3.6. Alkaline–Acid and Neutral–Acid Protein Extraction Followed by Enzymatic Digestion

The conventional method for protein extraction, which is alkaline solubilization followed by acidic precipitation, was also evaluated in this study. However, since strong alkaline conditions may lead to protein denaturation [12], a milder alternative was also tested, involving solubilization of proteins in a neutral environment followed by acid precipitation. Even though both the conventional and the direct enzyme-assisted digestion routes ultimately produce peptide hydrolysates, they should be regarded as distinct processing workflows rather than interchangeable procedures.

**Figure 4:**
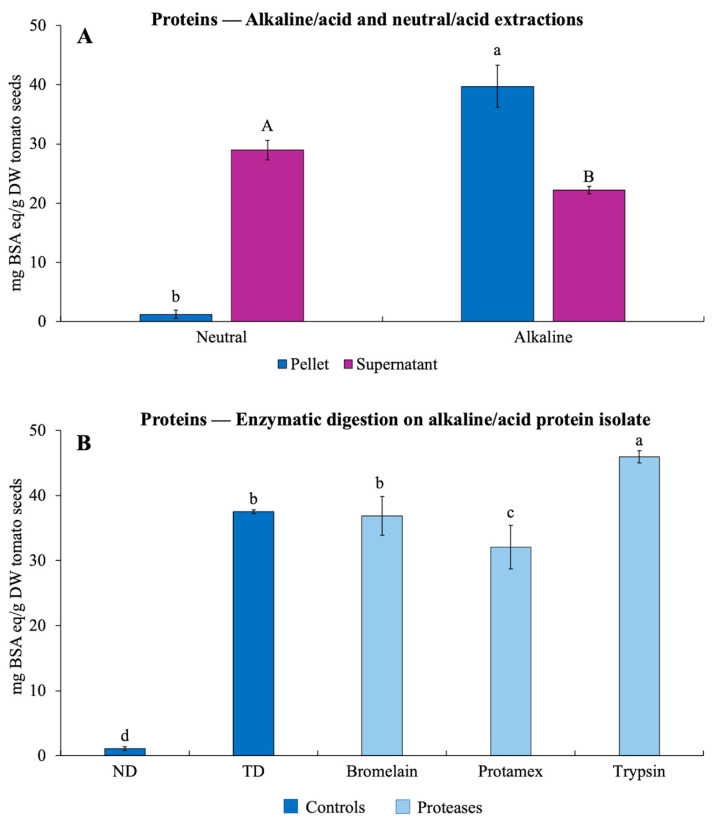
Protein content (mg BSA eq/g DW) of (**A**) protein isolates (resuspended pellets) and supernatants after alkaline or neutral solubilization followed by acidic precipitation, and (**B**) of digestates of alkaline/acid protein isolates following enzymatic digestion at 60 °C for 2 h. In panel (**A**), lowercase and capital letters indicate statistically significant differences among samples of protein isolates (resuspended pellets) or among supernatants, respectively, determined by ANOVA test followed by post hoc Tukey HSD test (*p* < 0.05). In panel (**B**), letters indicate statistically significant differences among samples determined by ANOVA test followed by post hoc Tukey HSD test (*p* < 0.05). BSA, bovine serum albumin; DW, dry weight; ND, non-digested control; TD, thermally digested control. Data are the mean (n = 4) ± SD.

Alkaline solubilization was more effective than neutral solubilization for protein recovery, yielding 39.72 mg BSA eq/g DW and 1.24 mg BSA eq/g DW, respectively, in the resuspended protein pellet. In both conditions, a fraction of proteins/peptides remained unprecipitated, with concentrations of 22.21 mg BSA eq/g DW in the alkaline supernatant and 28.99 mg BSA eq/g DW in the neutral supernatant (Figure 4A). Previous studies have applied the conventional alkaline extraction approach to TS to obtain protein isolates [7,10]. As reported by Rana et al. [10], increasing the solubilization pH enhances protein recovery, a trend that was also confirmed in the present study (Figure 4A).

The alkaline/acid protein isolates were subsequently digested at 60 °C for 2 h using the previously selected proteases, following the procedure described in Section 2.2 and applied to raw TS. ND and TD controls were also included. Both protease and TD treatments significantly increased the protein content of the digestates compared to the ND control, with an average 23.3-fold increase (Figure 4B). As noted earlier, thermal treatment alone contributed to partial protein breakdown in the protein isolate. Trypsin was the only enzyme that significantly enhanced protein recovery compared to the TD control, reaching 45.96 mg BSA eq/g DW. Despite this improvement, the value remained significantly lower (55.4% reduction, Figure 4B) than that obtained for Trypsin-treated raw TS incubated at 60 °C (Figure 1B).

The comparison of peptide yields obtained through enzyme-assisted protein extraction directly applied to raw TS (Figure 1A and Figure 2A) with those shown in Figure 4B highlights the advantage of the former approach. On average, digestates produced using the seven tested proteases reached 104.14 mg BSA eq/g DW at 60 °C (Figure 1A), while a value of 100.96 mg BSA eq/g DW was obtained at 37 °C (Figure 2) when only the three selected proteases were considered. In contrast, enzymatic digestion of the alkaline/acid protein isolates yielded an average of only 38.28 mg BSA eq/g DW. Although the enzyme-assisted direct protein extraction of TS was performed at neutral pH, it proved more effective in hydrolysing proteins than the conventional alkaline/acid method.

These findings indicate that proteases can enhance protein recovery while avoiding the harsh alkaline conditions required in the conventional extraction process. Moreover, in both alkaline and neutral extractions followed by acidic precipitation, a fraction of proteins remained unprecipitated in the supernatant (Figure 4A), likely due to their low molecular weight. This observation supports the hypothesis that some of the extracted peptides (Figure 1A and Figure 2A) were generated within the seeds during tomato industrial processing; their small size likely hindered efficient precipitation, resulting in lower protein recovery.

Only a limited number of studies have investigated enzymatic digestion and bioactivity of the protein isolate. For example, Meshginfar et al. [11] enzymatically hydrolyzed protein fractions extracted from defatted dTS via alkaline treatment and reported enzyme ratio-dependent increases in both antioxidant and ACE-inhibitory activities.

Furthermore, while the conventional alkaline/acid method generally yields protein isolates composed mainly of large intact proteins [11], the enzyme-assisted direct protein extraction may produce low-molecular-weight peptides that typically exhibit higher bioactivity than intact proteins [17], a trend that was also confirmed in the present study (Figure 3A,B).

## 4. Conclusions

This study demonstrates that direct enzyme-assisted extraction, particularly using proteases, represents a promising and sustainable approach for recovering proteins/peptides from tomato seeds, by-products of tomato industrial processing. By optimizing key parameters, including raw material pre-treatment conditions, enzyme type, enzyme/substrate ratio, and incubation temperature, the most efficient and environmentally sustainable protocol was identified. Protease treatment generated digestates, including low-molecular-weight peptides with enhanced antioxidant and anti-tyrosinase activities, highlighting the advantages of this method that may allow greater control over the characteristics of the resulting peptides. Beyond enhancing protein recovery and functionality, direct enzyme-assisted protein extraction offers significant practical and environmental benefits. The use of milder extraction conditions reduces the risk of protein denaturation, thereby preserving peptide bioactivity. Moreover, avoiding a preliminary alkaline/acid extraction lowers chemical consumption and equipment requirements, resulting in a simpler and more sustainable process. Some enzymes may also be more cost-effective due to their microbial origin, such as Protamex, which generated peptides with enhanced antioxidant and anti-tyrosinase activities.

Overall, producing bioactive peptides through protease treatment may enable the further industrial valorisation of tomato seeds by transforming a by-product into a high-value resource. Moreover, the use of a green extraction approach reduces the environmental impact of tomato processing and supports circular economy and sustainability principles. However, further studies are still required to identify the specific bioactive peptides generated and to validate the scalability and industrial applicability of the process at pilot scale.

## Figures and Tables

**Table 1:** Protein, reducing sugar and total phenolic contents in tomato seed samples following two-step enzymatic digestion treatments.

Samples	Proteins (mg BSA eq/g DW)	Reducing Sugars (mg GLU eq/g DW)	Total Phenolics (mg GA eq/g DW)
TD (4 h)	54.29 ± 5.90 ^c^	5.79 ± 0.51 ^d^	1.91 ± 0.29 ^b^
Viscozyme + Bromelain	94.82 ± 4.22 ^b^	41.26 ± 4.39 ^b^	4.20 ± 0.40 ^a^
Viscozyme + Protamex	105.67 ± 8.92 ^ab^	36.84 ± 1.29 ^c^	4.18 ± 0.26 ^a^
Viscozyme + Trypsin	111.68 ± 12.20 ^a^	53.03 ± 3.14 ^a^	4.23 ± 0.47 ^a^

Notes: Each enzyme was added at 5% (*w*/*w*) E/S ratio and incubated at 60 °C for 2 h per step. Letters indicate statistically significant difference among samples for each compound determined by ANOVA test followed by post hoc Tukey HSD test (*p* < 0.05). BSA, bovine serum albumin; DW, dry weight; GA, gallic acid; GLU, glucose; TD, thermally digested control. Data are the mean (n = 4) ± SD.

## Data Availability

Data will be made available on Recherche Data Gouv and can be found at the following link: https://doi.org/10.57745/SIWI9L (accessed on 25 May 2026).

## Supplementary Materials

The following supporting information can be downloaded at: https://www.mdpi.com/article/10.3390/foods15111934/s1. Table S1: Biochemical characterization of tomato seed extracts after enzymatic treatment with 1% and 2% E/S ratio; Table S2: Protein content in defatted tomato seeds following 5% E/S enzymatic treatment; Figure S1: Mono-dimensional SDS-PAGE of tomato seed digestates following protease treatment.

## Abbreviations

The following abbreviations are used in this manuscript:

TSs	Tomato Seeds
dTSs	Defatted Tomato Seeds
E/S	Enzyme/Substrate Ratio
DW	Dry Weight
ND	Non-Digested Control
TD	Thermally Digested Control
BSA	Bovine Serum Albumin
GLU	Glucose
GA	Gallic Acid
AA	Ascorbic Acid
KA	Kojic Acid
