# Supplementary material for: Unlocking Bioactive, Peptide-Rich Extracts from Tomato Seeds Using Enzymatic-Assisted Extraction
Source: Foods. 2026 May 29;15(11):1934. doi: 10.3390/foods15111934 (PMC13256655; doi:10.3390/foods15111934)
Supplement: Supplementary file 1 [file foods-15-01934-s001.zip › Supplementary files rev1 pdf/Supplementary Figure S1_rev1.pdf]

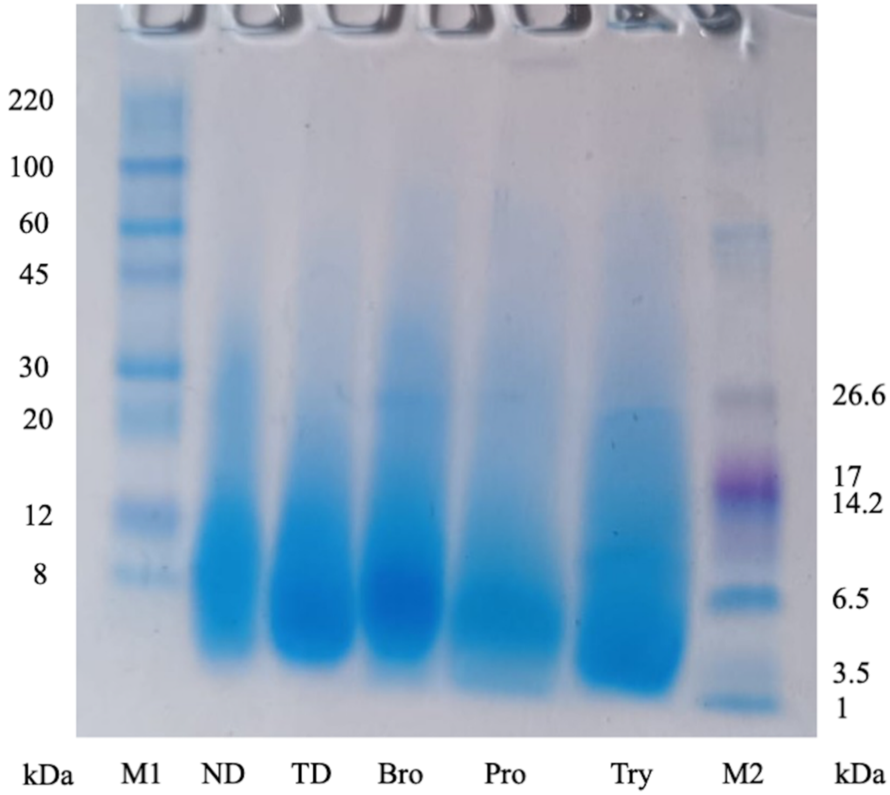

Figure S1: Mono-dimensional SDS-PAGE (8-16% w/v acrylamide) of tomato seed digestates following protease treatment at 37 °C for 2 h with Bromelain, Protamex and Trypsin 5% (w/w) E/S. Loading of the gel: digestates 20 μL; ND 25 μL; TD, 10 μL. The gel was stained with Coomassie ProBlue Safe Stain. M1, protein molecular weight marker 220-8.0 kDa; ND, not-digested control; TD, thermally-digested control; Bro, Bromelain; Pro, Protamex; Try, Trypsin; M2, protein molecular weight marker 26.6-1.0 kDa.
