# Supplementary material for: Unlocking Bioactive, Peptide-Rich Extracts from Tomato Seeds Using Enzymatic-Assisted Extraction
Source: Foods. 2026 May 29;15(11):1934. doi: 10.3390/foods15111934 (PMC13256655; doi:10.3390/foods15111934)
Supplement: Supplementary file 1 [file foods-15-01934-s001.zip › Supplementary files rev1 pdf/Supplementary Table S1_rev1.pdf]

Supplementary Table S1: Biochemical characterization of tomato seed extracts following enzymatic treatment with 1% and 2% (w/w) enzyme/substrate ratio (E/S).

**Samples**	**Proteins** (mg BSA eq/g DW)		**Reducing sugars** (mg GLU eq/g DW)		**Total phenolics** (mg GA eq/g DW)	
**Controls**						
ND	19.28 ± 1.16 $^{g\ F}$		1.11 ± 0.02 $^{g\ H}$		1.05 ± 0.09 $^{hi\ EF}$	
TD pH 7	49.15 ± 4.74 $^{ef\ DE}$		3.06 ± 0.24 $^{de\ EF}$		1.38 ± 0.13 $^{fgh\ EF}$	
TD pH 6	55.40 ± 3.69 $^{de\ D}$		2.29 ± 0.15 $^{efg\ FG}$		0.82 ± 0.06 $^{i\ F}$	
**Enzymes**	**1% (w/w) (E/S)**	**2%(w/w) (E/S)**	**1% (w/w) (E/S)**	**2% (w/w) (E/S)**	**1% (w/w) (E/S)**	**2% (w/w) (E/S)**
Alcalase	100.70 ± 10.40 ^{a}	98.18 ± 8.24 ^{A}	2.32 ± 0.39 ^{ef}	1.74 ± 0.16 ^{GH}	3.48 ± 0.52 ^{ab}	3.66 ± 0.73 ^{B}
Bromelain	80.48 ± 9.72 ^{b}	97.59 ± 15.54 ^{A}	3.80 ± 0.10 ^{cd}	3.46 ± 0.23 ^{E}	2.81 ± 0.31 ^{cd}	3.09 ± 0.57 ^{BC}
Neutrase	64.40 ± 5.87 ^{cd}	75.17 ± 12.20 ^{C}	1.79 ± 0.41 ^{fg}	1.73 ± 0.03 ^{GH}	2.32 ± 0.13 ^{de}	2.66 ± 0.28 ^{CD}
Pancreatin	79.13 ± 4.84 ^{b}	82.31 ± 7.98 ^{BC}	3.09 ± 0.60 ^{de}	3.25 ± 0.24 ^{E}	3.82 ± 0.25 ^{a}	4.32 ± 0.47 ^{A}
Papain	66.46 ± 11.87 ^{cd}	89.53 ± 11.25 ^{AB}	4.50 ± 0.71 ^{c}	5.43 ± 0.15 ^{D}	1.84 ± 0.36 ^{ef}	2.23 ± 0.36 ^{D}
Protamex	82.82 ± 6.15 ^{b}	87.92 ± 1.71 ^{ABC}	2.10 ± 0.32 ^{efg}	1.74 ± 0.04 ^{GH}	2.82 ± 0.43 ^{c}	2.56 ± 0.46 ^{CD}
Trypsin	76.77 ± 5.42 ^{bc}	97.58 ± 5.38 ^{A}	8.99 ± 1.41 ^{a}	14.20 ± 0.40 ^{A}	3.22 ± 0.63 ^{bc}	3.31 ± 0.28 ^{B}
Celluclast	46.68 ± 9.77 ^{ef}	44.74 ± 6.99 ^{DE}	3.85 ± 0.29 ^{cd}	3.26 ± 0.28 ^{E}	1.44 ± 0.13 ^{fgh}	1.44 ± 0.31 ^{E}
Pectinex Ultra SP-L	47.39 ± 6.00 ^{ef}	41.82 ± 3.42 ^{E}	7.79 ± 0.09 ^{b}	11.56 ± 0.48 ^{C}	1.31 ± 0.04 ^{ghi}	1.26 ± 0.06 ^{EF}
Pectinex XXL	43.42 ± 8.84 ^{ef}	48.25 ± 7.14 ^{DE}	3.27 ± 0.38 ^{de}	3.54 ± 0.20 ^{E}	1.41 ± 0.14 ^{fgh}	1.44 ± 0.15 ^{E}
Pentopan	37.40 ± 6.36 ^{f}	38.67 ± 2.30 ^{E}	2.92 ± 0.16 ^{def}	2.97 ± 0.18 ^{EF}	1.55 ± 0.10 ^{fg}	1.48 ± 0.07 ^{E}
Viscozyme	46.69 ± 4.21 ^{ef}	47.94 ± 8.54 ^{DE}	9.25 ± 1.86 ^{a}	12.81 ± 1.76 ^{B}	1.38 ± 0.07 ^{fgh}	1.41 ± 0.07 ^{E}

Notes: Digestion was performed at 60°C for 2 h. ND, TD pH 7 and TD pH6 values are the same as reported in Figure 1 and are here again repeated for clarity. Lowercase letters indicate statistically significant difference among samples with 1% E/S ratio and their controls determined by ANOVA test followed by post-hoc Tukey HSD test ($p < 0.05$). Capital letters indicate statistically significant difference among samples with 2% E/S ratio and their controls determined by ANOVA test followed by post-hoc Tukey HSD test ($p < 0.05$). BSA, bovine serum albumin; DW, dry weight; GA, gallic acid; GLU, glucose; ND, non-digested control; TD, thermally digested control. Data are the mean (n=4) ± SD.
