# Supplementary material for: Unlocking Bioactive, Peptide-Rich Extracts from Tomato Seeds Using Enzymatic-Assisted Extraction
Source: Foods. 2026 May 29;15(11):1934. doi: 10.3390/foods15111934 (PMC13256655; doi:10.3390/foods15111934)
Supplement: Supplementary file 1 [file foods-15-01934-s001.zip › Supplementary files rev1 pdf/Supplementary Table S2_rev1.pdf]

Supplementary Table S2: Protein content in defatted tomato seeds samples following 5% (w/w) E/S enzymatic treatment.

**Samples**	**Proteins** (mg BSA eq/g DW dTS)
ND	12.14 ± 2.09 [c]
TD	43.05 ± 1.91 [b]
Protamex	112.27 ± 18.86 [a]
Trypsin	110.27 ± 9.88 [a]

Notes: Digestion was performed at 60°C for 2 h. Letters indicate statistically significant difference among samples determined by ANOVA test followed by post-hoc Tukey HSD test ($p < 0.05$). BSA, bovine serum albumin; DW, dry weight; ND, non-digested control; TD, thermally digested control; dTSs, defatted tomato seeds. Data are the mean (n=4) ± SD.
